# 3‐Hydroxyisobutyryl‐CoA hydrolase deficiency in an infant with developmental delay and high anion gap acidosis

**DOI:** 10.1002/ccr3.4528

**Published:** 2021-07-23

**Authors:** Hedyeh Saneifard, Asieh Mosallanejad, Aida Fallahzadeh, Ali Sheikhy

**Affiliations:** ^1^ Mofid Children's Hospital Shahid Beheshti University of Medical Sciences Tehran Iran; ^2^ Imam Hosein Medical Center Shahid Beheshti University of Medical Sciences Tehran Iran; ^3^ Research Department Tehran Heart Center Tehran University of Medical Sciences Tehran Iran; ^4^ Non‐Communicable Diseases Research Center (NCDRC) Endocrinology and Metabolism Population Sciences Institute Tehran University of Medical Sciences Tehran Iran

**Keywords:** 3‐hydroxyisobutyryl‐CoA, acidosis, developmental disability, dystonia

## Abstract

Due to the rarity of this disorder, paying attention to diagnostic clues is important. Low valine formula seems to be effective in improvement of patient's symptoms. Prevention of consanguineous marriage is the best way to prevent this disease.

## Graphical abstract

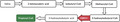

## 1 INTRODUCTION

3‐hydroxyisobutyryl‐CoA hydrolase (HIBCH) deficiency is a recently described, rare inborn error of valine's metabolism. We report an 11‐month‐old Iranian girl presenting with developmental delay and high anion gap metabolic acidosis. Upon whole‐exome sequencing, she was diagnosed with HIBCH deficiency as compound heterozygosity and subjected to specific dietary treatment.

3‐hydroxyisobutyryl‐CoA hydrolase (HIBCH) deficiency is a recently described, rare inborn error of valine's metabolism that results from mutations in the *HIBCH* genes. Its incidence is as high as 1 in 130,000 individuals.^1^ HIBCH is a mitochondrial enzyme responsible for specific hydrolysis of 3‐hydroxyisobutyryl‐CoA, the fifth step of valine catabolism, and the hydrolysis of beta‐hydroxypropionyl‐CoA, an intermediate in a minor pathway of propionate metabolism.^2^ The pathogenic effect of HIBCH deficiency is due to accumulation of toxic valine metabolites which potentially interfere with mitochondrial enzymes.^3^ HIBCH deficiency is an autosomal recessive disorder which is characterized by seizure starting in infancy, developmental delay, Leigh‐like changes in the basal ganglia, dystonia, congenital malformations, microcephaly, episodes of ketoacidosis, and encephalopathy.^4^ Only few cases have been reported worldwide, and thus, the characteristics, genes, and optimal treatment are not well defined. Here, we report an 11‐month‐old infant with seizure, dystonia, high anion gap acidosis, and developmental delay who was admitted to our center.

## 2 CASE REPORT

### 2.1 Clinical course and diagnostic work‐up

**FIGURE 1:**
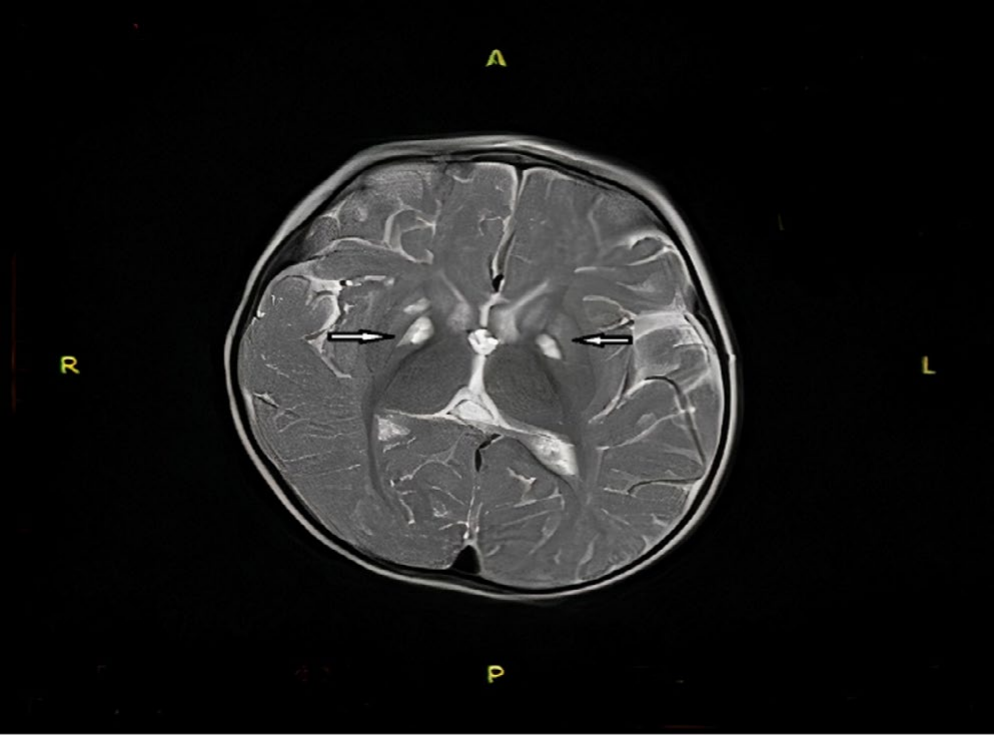
Brain MRI showed symmetrical hypersignal abnormality in the basal ganglia (white arrows)

The patient was an 11‐month‐old Iranian girl, the second child of healthy, unrelated parents. Her mother was a multigravida woman with a history of one abortion. She was admitted to our hospital for respiratory distress, seizure, nausea, and vomiting. According to her history, she had developmental delay as she was unable to sit unsupported. On physical examination, she had dystonia. Laboratory findings showed high anion gap metabolic acidosis (PH = 7.09; HCO3 = 3.4 mEq/L; PCO2 = 11 mmHg; anion gap = 24) on her first venous blood gas (VBG) and 3+ ketone in urine analysis. Complete blood count (CBC) was normal except for an increased in white blood cell (WBC = 18,800; neutrophil = 80%). Serum creatinine, blood urea nitrogen (BUN), sodium, potassium, calcium, phosphorus, triglyceride, and cholesterol tests were normal. Liver function tests were normal (SGOT = 29 IU/L, SGPT = 17 IU/L). Due to severe respiratory distress, she was intubated and admitted to intensive care unit (ICU). Sepsis work‐up was done which was negative (negative blood culture on day 1 and 7). Phenytoin, levetiracetam, and vitamin B6 were administered for her seizure's management. Due to suspicion to metabolic disease, metabolic work‐up was done. Lactate levels were normal (17.5 mg/dl, normal range: 4.5–20 mg/dl), whereas serum ammonia (1.05 μg/ml, normal range: 0.17–0.87 μg/ml) and pyruvate (1.2 mg/dl, normal range: 0.3–0.9) levels were slightly increased. Serum and urine amino acid chromatography (HPLC) was normal. Acylcarnitine profile and urine organic acid profile were normal. In cardiologic evaluation, a mild left ventricular hypertrophy (LVH) was reported. Brain MRI showed symmetrical hypersignal abnormality in the basal ganglia (caudate and lentiform nucleolus) (Figure 1).

### 2.2 Molecular genetic analysis

Due to high suspicion of metabolic disease and normal metabolic work‐up, whole‐exome sequencing was performed. Agilent's SureSelect Human All Exon V6 kit was used for enrichment. An end‐to‐end in‐house bioinformatics pipeline including base calling, alignment of reads to *GRCh37/hg19* genome assembly, primary filtering out of low‐quality reads and probable artifacts, and subsequent annotation of variants, was applied. Evaluation was focused on coding exons along with flanking ±20 intronic bases. All pertinent inheritance patterns were considered. In addition, provided family history and clinical information were used to evaluate eventually identified variants. Targeted sequencing was performed on both DNA strands of the relevant HIBCH region. The reference sequence was *HIBCH*: NM_014362.3

A heterozygous likely pathogenic variant and heterozygous variant of uncertain significance were identified in the HIBCH gene (Table 1). In continue, we examined parents genetic. The reference sequence in mother was *HIBCH*: Chr2(GRCh37): g.191110882G>A NM_014362.3: c.807C>T, p. (Asn269Asn). Furthermore, the genetic result in the father was *HIBCH*: Chr2(GRCh37): g.191159365A>C NM_014362.3: c.220‐9T>G (Table 2).

**TABLE 1:** Variant description based on Alamut Batch

Gene	Variant coordinates	Zygosity	Type and classification
*HIBCH*	Chr2(GRCh37):g.191110882G>A NM_014362.3:c.807C>T p. (Asn269Asn) Exon 10	Het	Synonymous Uncertain significance (class 3)
*HIBCH*	Chr2(GRCh37):g.191159365A>C NM_014362.3:c.220‐9T>G Intron 3	Het	Substitution Likely pathogenic (class 2)

**TABLE 2:** Variant description of parents

	Gene	Variant coordinates	Zygosity	Type and classification
Mother	*HIBCH*	Chr2(GRCh37): g.191110882G>A NM_014362.3: c.807C>T p. (Asn269Asn) Exon 10	Het	Synonymous Uncertain significance (class 3)
Father	*HIBCH*	Chr2(GRCh37): g.191159365A>C NM_014362.3: c.220‐9T>G Intron 3	Het	Substitution Likely pathogenic (class 2)

According to the results from parental genetics, we can consider this medical genetic in the reported infant as ‘compound heterozygosity’, thus diagnosis of 3‐hydroxyisobutyryl‐CoA hydrolase deficiency confirmed.

### 2.3 Treatment and results

According to the pathogenesis of HIBCH deficiency, a low valine diet was prescribed, which include dietary restriction of proteins as an amount of 1.7 g/Kg/day. To control seizure's attack, topiramate, levetiracetam, and clonazepam were prescribed. Supplementation with carnitine, vitamin E, vitamin C, and co‐Q10 was done, and N‐acetyl cysteine (200 mg/day) was prescribed due to their antioxidant effects. On her follow‐up visits, the developmental status was progressed and in age 16 months old she was able to sit, seizures were controlled, and no acidotic attack was repeated anymore.

## 3 DISCUSSION

In this article, we reported a 11‐month‐old girl with developmental delay, dystonia, high anion gap metabolic acidosis and seizure attacks and normal metabolic laboratory results, with the final diagnosis of 3‐hydroxyisobutyryl‐CoA hydrolase (HIBCH) deficiency according to whole‐exome sequence (WES).

**FIGURE 2:**
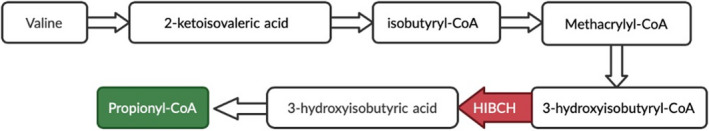
Summary of valine metabolism, highlighting the role of 3‐hydroxyisobutyryl‐CoA hydrolase (HIBCH)

3‐hydroxyisobutyryl‐CoA hydrolase deficiency is an autosomal recessive disorder caused by mutations in HIBCH genes and characterized by severe psychomotor delay, progressive neurodegeneration, recurrent metabolic attacks with intercurrent illness, brain lesions in basal ganglia, and seizure disorder. Although manifestations of HIBCH deficiency overlap with other mitochondrial disorders, the management is different and dietary valine and protein restriction appears to be beneficial in treatment. Valine is one the of the essential branched‐chain amino acids (BCAA), which are present in all protein‐containing foods, such as dairy products, meat, egg, and whey. Valine is metabolized in the mitochondria via the enzymatic pathway including HIBCH. First, valine converts to methacrylyl‐CoA and then to 3‐hydroxyisobutyryl‐CoA. In the next step, HIBCH converts 3‐hydroxyisobutyryl‐CoA to 3‐hydroxyisovaleric acid and finally to the end product propionyl CoA^5^ (Figure 2). The pathogenesis of HIBCH deficiency is explained through the accumulation of toxic valine metabolites especially methacrylyl‐CoA compounds in the liver, kidneys, and brain. Moreover, methacrylyl‐CoA accumulates in mitochondria and causes a secondary mitochondriopathy through reactions with thiol‐containing compounds and also depletes mitochondrial pools of cysteine, glutathione, CoA, or lipoic acid.^3^, ^6^

Few cases of HIBCH deficiency have been reported so far. The first case of HIBCH deficiency was a male infant with multiple physical malformations (dysmorphic facial features, multiple vertebral anomalies, tetralogy of Fallot, and agenesis of the cingulate gyrus and corpus callosum) as well as failure to thrive and marked hypotonia, born to parents who were cousins, in 1982.^7^ Second case of HIBCH deficiency was a male infant and the first child of nonconsanguineous parents, with developmental delay, ataxia, dysmetria, tremor, and metabolic acidosis as were seen in our case.^4^ There is another case from Iran, who was a 4‐year‐old boy of nonconsanguineous parents with hyperactive deep tendon reflex, general hypotonia, ataxia, dysmetria, developmental delay including inappropriate walking, talking, and growth parameters and recurrent attacks following febrile diseases with symptoms including weakness, myoclonus, and eye nystagmus.^8^ As was seen in our case, the brain MRI showed bilateral high signal lesions in globus pallidus (Leigh‐like syndrome).

There is another case of two Pakistani brothers born to distantly related parents presenting with progressive neurodegenerative disorder and atrophy in globus pallidus on MRI.^9^ They had developmental regression, progressive hypotonia, recurrent generalized seizures, and also persistent vomiting despite the fundoplication. They had positive family history of neurologic disorders and neuromuscular conditions, which was notable. Contrary to them, our patient's parents did not have a consanguineous marriage.

Most recent case was a 1‐year‐old female infant of consanguineous Turkish parents, with developmental delay, axial hypotonia, and metabolic acidosis and brain MRI lesion suggestive for mitochondrial disorder.^10^ Another case of a 6‐year‐old Chinese girl was also reported,^11^ presented with exercise‐induced dystonia, elevated blood ammonia level, and MRI abnormalities. Notably, all cases described above have similar clinical symptoms specially dystonia and abnormalities in basal ganglia in MRI, as were seen in our case. Thus, it is essential that HIBCH deficiency should be kept in mind in any patient with hypotonia, dystonia, developmental delay, and Leigh‐like syndrome.

## 4 CONCLUSION

3‐hydroxybutyryl‐CoA hydrolase (HIBCH) deficiency, a valine catabolism disorder, should be considered in the differential diagnosis of a patient with developmental delay, dystonia, and high anion gap metabolic acidosis and Leigh‐like syndrome. Due to the rarity of this disorder, paying attention to diagnostic clues is important. ‘Compound heterozygosity’ is a rare condition which should be considered in patients with more than one pathogenic foundlings in WES reports; consequently, parents should be examined in these cases. Low valine formula seems to be effective in improvement of patient's symptoms. Prevention of consanguineous marriage is the best way to prevent this disease.

## CONFLICT OF INTEREST

None declared.

## AUTHOR CONTRIBUTIONS

HS and AM contributed in data gathering and patient's diagnosis and treatment. AF and AS contributed in drafting and revision of the main manuscript.

## ETHICAL APPROVAL

Written informed consent was obtained from the parents. The study protocol was approved by the institute's committee on human research.

## Data Availability

The data that support the findings of this study are available from the corresponding author upon reasonable request.
